# ARPE-19—A Stable Cell Line Expressing a Variant of Unknown Significance in the *NPC1* Gene

**DOI:** 10.3390/genes17030288

**Published:** 2026-02-27

**Authors:** Beatriz Monteiro, Maria Inês Peixoto, Juan Darío Ortigoza-Escobar, Mariana Alves, Ana Catarina Sandiares, Mariana Gonçalves, Luciana Vaz Moreira, Maria Francisca Coutinho, Liliana Matos, Sandra Alves, Marisa Encarnação

**Affiliations:** 1 Research and Development Unit, Department of Human Genetics, National Institute of Health Doutor Ricardo Jorge, 4000-055 Porto, Portugal; beatrizsofiamonteiro2004@gmail.com (B.M.); m.ines.peixoto@insa.min-saude.pt (M.I.P.); luciana.moreira@insa.min-saude.pt (L.V.M.); francisca.coutinho@insa.min-saude.pt (M.F.C.); liliana.matos@insa.min-saude.pt (L.M.);; 2 Department of Genetics and Biotechnology, School of Life and Environmental Sciences, University of Trás-os-Montes and Alto Douro, 5000-801 Vila Real, Portugal; 3 Movement Disorders Unit, Pediatric Neurology Department, Institut de Recerca Hospital Sant Joan de Déu Barcelona, 08950 Barcelona, Spain; 4 European Reference Network for Rare Neurological Diseases (ERN-RND), 08035 Barcelona, Spain; 5 U-703 Centre for Biomedical Research on Rare Diseases (CIBER-ER), Instituto de Salud Carlos III, 08034 Barcelona, Spain; 6 Institute of Biomedicine (iBiMED), Department of Medical Sciences, University of Aveiro, 3810-193 Aveiro, Portugal; 7 Center for the Study of Animal Science, Instituto de Ciências, Tecnologias e Agroambiente (CECA-ICETA), University of Porto, Praça Gomes Teixeira, Apartado 55142, 4051-401 Porto, Portugal; 8 Associate Laboratory for Animal and Veterinary Sciences (AL4AnimalS), Faculdade de Medicina Veterinária, Avenida da Universidade Técnica, 1300-477 Lisboa, Portugal

**Keywords:** lysosomal storage diseases, Niemann–Pick disease type C, variants of unknown significance, cellular model, functional characterization

## Abstract

Background: Niemann–Pick type C is a lysosomal storage disorder that results from pathogenic variants in the *NPC1* gene or in some cases from *NPC2* pathogenic alterations. The disease presents a remarkable clinical variability that in some cases resembles common diseases, often resulting in a diagnostic odyssey or at least delaying proper diagnosis. In addition, the *NPC1* gene is highly polymorphic, and consequently, when missense variants are identified after gene sequencing, accurate classification of their pathogenicity is essential to ensure appropriate access to available therapies and to provide reliable genetic counseling. Objectives: To get insights into the pathogenicity of a novel variant in NPC1, p.Cys800Ser, we created stable cell lines expressing this variant, in parallel with cell lines expressing the NPC1 wild-type and NPC1 pathogenic variants. Methods: We leveraged an isogenic cell line in which the *NPC1* gene was knocked down and subsequently infected it with retroviruses carrying NPC1-WT and NPC1 variants C-terminally fused with an mNeonGreen tag. Three different NPC1 variants were included in this study: two known pathogenic variants, p.Ala1035Val and p.Pro1007Ala, and the novel p.Cys800Ser, whose significance was unknown. Results: We observed in the stable cell line expressing NPC1 p.Cys800Ser that the mutated NPC1 protein is transported to the lysosome similarly to the p.Pro1007Ala variant and affects lysosomal distribution. Conclusions: Using this approach, we could analyze the pathogenicity of each variant separately and these cell lines could be used for personalized medicine-based approaches and multi-omic studies.

## 1. Introduction

Niemann–Pick disease type C (NPC; OMIM# 257220) is a monogenic, autosomal recessive disorder that belongs to the group of lysosomal storage diseases (LSDs). It is caused by pathogenic variants in the *NPC1* gene (in more than 95% of cases) or, less frequently, in the *NPC2* gene [1]. NPC shows high clinical variability, often with visceral, neurological, and psychiatric symptoms that overlap with other conditions, leading to significant delays in diagnosis and limited access to available therapies [2]. Furthermore, the *NPC1* gene is highly polymorphic, with a significant proportion of variants classified as of uncertain significance (VUS); remarkably, it is estimated that around 1/3 of the variants reported in the ClinVar database (https://www.ncbi.nlm.nih.gov/clinvar) fall into this category. Upon the identification of a novel variant(s) in a patient with clinical suspicion of NPC, checking its frequency in large databases and performing in silico analysis are the initial steps to be performed. Both will help in variant prioritization for posterior experimental validation (in vitro and in vivo approaches), ultimately allowing their classification, according to The American College of Medical Genetics and Genomics (ACMG) guidelines [3].

A multitude of in silico tools have been developed in recent decades to predict the pathogenicity of variants and, more recently, the deep learning tool AlphaMissense combines both structural context and evolutionary conservation, predicting the pathogenicity of all possible single amino acid changes at a given position [4]. Although AlphaMissense predictions can significantly reduce the number of variants classified as VUS, its utility decreases when a variant’s pathogenicity score falls into the ambiguous “intermediate” range [5]. In these cases, the predicted scores do not offer enough certainty to confidently reclassify the variant, requiring the use of in vitro functional assays to gather the empirical evidence needed for a definitive clinical interpretation.

Some in vitro studies for investigating VUS include saturation genome editing (SGE), which enables in-depth functional evaluation of disease-associated genes and variants by generating all possible single-nucleotide variants (SNVs) within a given coding region [6]. This high-throughput functional characterization was applied to the *NPC1* gene, with 410 of 706 assayed missense mutations being classified as deleterious, suggesting that NPC1 is very sensitive to genetic perturbation [6]. Other more targeted approaches include in vitro analysis of patient-derived samples such as patients’ skin fibroblasts or, more recently, induced pluripotent stem cells (iPSCs), but they have technical and interpretative limitations. Additionally, in cases where VUS are found in compound heterozygosity with a known pathogenic variant, functional studies in patient-derived cells may be inconclusive. Therefore, there is a need for complementary cellular systems that allow a more accurate assessment of the pathogenicity of these variants. In this context, the aim of this work was to contribute to the functional characterization of a novel NPC1 variant, using a robust in vitro cellular model. Here, we used a cellular model whose *NPC1* gene was silenced (NPC1^−/−^ ARPE-19: an immortalized retinal pigment epithelial cell line), which was then transduced with retroviral particles encoding different NPC1 variants tagged with fluorescence. Subcellular localization assays were performed, with a particular focus on the intracellular trafficking of the NPC1 protein to the lysosome and the lysosome positioning

In addition to the stable cell line expressing the VUS p.Cys800Ser, other stable cell lines were also generated for control purposes, one expressing the NPC1 wild-type protein and two others expressing NPC1 proteins harboring well-characterized disease-causing variants: a missense variant associated with a severe NPC phenotype (p.Ala1035Val) [7,8] and a missense variant associated with a later-onset phenotype that is less severe (p.Pro1007Ala) [7,9]. The results obtained by fluorescence microscopy, aiming to visualize the localization of the NPC1 protein tagged with mNeonGreen in its wild-type version and with the aforementioned variants, showed that our target variant has a distribution pattern similar to that of the p.Pro1007Ala variant and more distinct from that observed for the p.Ala1035Val variant. Our data suggests that, in cells transduced with the p.Cys800Ser variant, intracellular trafficking of the NPC1 protein to the lysosome occurs, even though a disturbed lysosomal localization was observed at least partially. These results, although not conclusive, contribute to a deeper understanding of the phenotype observed in the patient carrying this variant, further highlighting the modeling value of these ARPE-19 stable cell lines.

## 2. Materials and Methods

### 2.1. Cell Lines

Spontaneously arising retinal pigment epithelia cell line (ARPE-19) and *NPC1*^−/−^ ARPE-19 cells were provided by Andrea Ballabio’s group at the Telethon Institute of Genetics and Medicine (Pozzuoli, Italy), and cultured in DMEM F12 and GlutaMAX (Gibco, ThermoFisher Scientific, Waltham, MA, USA) with 10% FBS at 37 °C with 5% CO_2_.

For the generation of γ-retroviral particles carrying the various *NPC1* constructs, a human embryonic kidney 293T (HEK293T) cell line was used, which was kindly provided by John Neidhart at the Carl von Ossietzky University of Oldenburg (Oldenburg, Germany).

### 2.2. Plasmids

pBABE-puro + NPC1-mNeonGreen was a kind gift from Kartik Chandran. pCMV-VSV-G and pUMVC were both gifts from Bob Weinberg (Addgene plasmids #8454 and #8449, respectively).

### 2.3. Antibodies

Primary antibodies were used: mouse α-Actin(C-2), 1:5000 (sc-8432, Santa Cruz Biotechnology, Dallas, TX, USA); mouse α-LAMP1(H4A3), 1:200 imaging (sc-20011, Santa Cruz Biotechnology, Dallas, TX, USA); and rabbit α-NPC1, 1:5000 (a134113, Abcam, Cambridge, UK).

The following secondary antibodies were used: goat α-mouse IgG-HRP, 1:40,000 (sc-2005, Santa Cruz Biotechnology, Dallas, TX, USA); mouse α-rabbit IgG-HRP, 1:40,000 (sc-2357, Santa Cruz Biotechnology, Dallas, TX, USA); and AlexaFluor^TM^ 594 goat α-mouse IgG(H + L), 1:500 (A-11032, Invitrogen, Waltham, MA, USA).

### 2.4. Site-Directed Mutagenesis

The constructs containing the analyzed variants (p.Cys800Ser, p.Pro1007Ala and p.Ala1035Val) were generated using the QuikChange Lightning Site-Directed Mutagenesis Kit (Agilent Technologies, Santa Clara, CA, USA) using the pBABE-puro + NPC1-mNeonGreen construct as a template. The primers used to introduce the variants are available upon request. The success of each mutagenesis was confirmed by Sanger sequencing.

### 2.5. Generation of Stably Transduced NPC1^−/−^ ARPE-19 Cells

Retroviruses packaging the transgenes were produced by triple transfection in the human embryonic kidney 293T (HEK293T) cell line. The cells were cultured in 12-well plates until they reached approximately 70% confluency. Cells were then transfected with plasmid DNA mixes comprising the transfer vector (pBABE-puro + NPC1-mNeonGreen), packaging (pUMVC) and envelope (pCMV-VSV-G) in a 3:2:1 ratio, using room-temperature FuGENE^®^ HD transfection reagent (Promega, Madison, WI, USA). At 16 h post-transfection, 1 mL viral collection medium (VCM; 20 μM HEPES in DMEM supplemented with 10% FBS) was added to each well. The virus-rich medium (VRM) was then collected in a Biosafety Level 3 laboratory at 32 h and 56 h after adding the VCM and filtered through a 0.45 μm filter.

For the retroviral transduction, NPC1^−/−^ ARPE-19 cells were grown on 6-well plates to approximately 80% confluency and transduced using 500 μL of filtered VRM mixed with 500 μL DMEM/F-12 (Gibco^TM^, Grand Island, NY, USA) with 16 μg/mL polybrene (Milipore Sigma, Merck, Darmstadt; Germany) (the final concentration in the wells was 8 μg/mL). Transduced cell populations were selected with 1 μg/mL puromycin-containing medium for 5–6 days and replaced every 48 h.

### 2.6. Immunofluorescence Assays

The retrovirally transduced cells were seeded in 8-well Lab-Tek II chamber slides (Nunc^TM^, Roskilde, Denmark) and processed as previously described for subsequent immunofluorescence assays [10,11]. Images were obtained with the Zeiss LSM 880 confocal microscope (Zeiss, Oberkochen, Germany) using a 63× oil objective.

### 2.7. Western Blot

ARPE-19 cells were pelleted and lysed with RIPA buffer. To visualize the NPC1 and actin proteins, 10 µg of total protein was mixed with NuPAGE LDS Sample Buffer and NuPAGE Samples Reducing Agent (Invitrogen, Waltman, MA, USA), denatured at 70 °C for 10 min and loaded on Mini-PROTEAN^®^ Stain-free 4–15% precast gels (Bio-Rad, Hercules, CA, USA), according to the manufacturer instructions. The blot was performed at 4 °C for 45 min using a 300 mA current. After blocking in 5% BSA in PBS with 0.05% Tween-20 for 1 h at room temperature, the antibody was incubated overnight at 4 °C. After, the membranes were washed and incubated with the corresponding secondary antibodies for 45 min. Membranes were rinsed with PBS with 0.05% Tween-20 and images were acquired on a ChemiDoc^TM^ XRS+ imaging system (Bio-Rad, Hercules, CA, USA).

### 2.8. Image Processing

Fluorescence microscopy images were processed using ZEN Black (Zeissand Fiji (version 2.9.0, open source) software).

### 2.9. Colocalization Quantification

The LAMP1 and NPC1-mNeonGreen signals’ cross-correlation was quantified using the Coloc plugin (Fiji software, version 2.9.0) by selecting 20 regions of interest for each condition.

### 2.10. Statistical Analysis

Statistical analysis between groups was performed using GraphPad Prism Software (version 8.0.1, Boston, MA, USA) and we used one-way analysis of variance followed by Tukey’s multiple-comparison test.

## 3. Results

In the present study, we investigated the impact of an *NPC1* VUS on protein stability, intracellular trafficking and lysosomal positioning in order to categorize the variant and help with patient molecular diagnosis.

The variant p.Cys800Ser (c.2399G>C) under analysis in this study was identified in compound heterozygosity with a pathogenic *NPC1* variant (p.Ala521Ser- [12]) in a 14-year-old male patient after whole-exome sequencing (WES). Overall, the patient did not exhibit the most frequent NPC symptoms such as hepatosplenomegaly and vertical supranuclear gaze palsy, but presented with tremors, myoclonus, and borderline intellectual capacity (global cognitive index 73). The patient was first referred for WES for these unspecific symptoms, and after the identification of biallelic variants in the *NPC1* gene, the biomarker N-palmitoyl-O-phosphocholineserine (PPCS) [13], also known as lysoSM-509, was analyzed but yielded normal results. Plasma oxysterol analysis revealed 3β,5α,6β-cholestanetriol concentrations within the reference range (6.0–6.4 ng/mL; reference 0.5–8.0 ng/mL), and serum chitotriosidase activity was within normal limits (29.5–40.0 nmol/h/mL; reference 17.0–211.0 nmol/h/mL). Additionally, filipin staining performed in cultured fibroblasts showed only rare cells with perinuclear accumulation of unesterified cholesterol after low-density protein (LDL) loading, a finding considered inconclusive for Niemann–Pick disease type C. Due to the borderline intellectual quotient, an Array Comparative Genomic Hybridization (CGH) was done and revealed that the patient is also a carrier of a deletion in the chromosome 15, del15q11.2 (arr[GRCh37] 15q11.2(22765628_23179948)x1), inherited from the mother. This deletion comprises four highly conserved genes: *TUBGCP5*, *NIPA1*, *NIPA2* and *CYFIP1*. Patients reported in the literature with this deletion exhibit some degree of learning difficulties, delayed development and/or behavioral problems. Thus, it is reasonable to assume that the del15q11.2 carrier status of the patient here reported underlies/explains his borderline intellectual quotient, but not the tremors or myoclonus. Interestingly, myoclonus is an observed symptom in some NPC patients, being considered as a subtle and easily overlooked manifestation of the disease [14].

Thus, due to the presence of the biallelic *NPC1* variants in this patient, and the fact that the other variant is already reported as disease-causing, we further investigated the p.Cys800Ser variant using both in silico and in vitro tools.

### 3.1. In Silico Analysis of the p.Cys800Ser Variant

**Figure 1:**
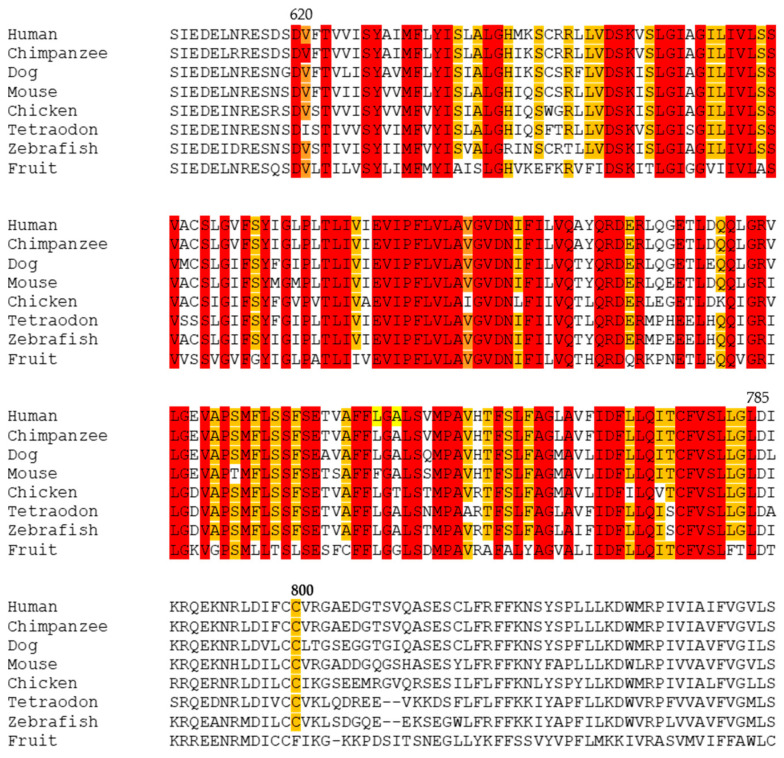
Interspecies sequence alignment of the NPC1 protein around the sterol-sensing domain and the amino acid residue on position 800. The sterol-sensing domain appears in a group of proteins related to cholesterol metabolism and transport, and in the case of human NPC1 protein encompasses the amino acids 620 and 785. The residues are shaded based on their levels of conservation in the alignment (in orange conserved in 7/8 species and red conserved in 8/8 species. The variant p.Cys800Ser is conserved in 7/8 species. The alignment was done using CLUSTALW (https://www.genome.jp/tools-bin/clustalw, accessed on 15 January 2026) and the amino acid sequences from the different species were extracted from the Ensembl databases (https://www.ensembl.org/index.html, accessed on 15 January 2026).

The variant p.Cys800Ser is not reported in the HGMD (https://my.qiagendigitalinsights.com/) or gnomAD databases (https://gnomad.broadinstitute.org/). Besides this, the amino acid alteration occurs near a conserved domain—the sterol-sensing domain—and the 800 residue itself is quite conserved (Figure 1). Notably, a disease-causing variant affecting the same amino acid residue, p.Cys800Arg, has been reported, resulting from a different nucleotide change (c.2398T>C) [15].

Later, we used AlphaMissense [4], a deep learning model that builds on the protein structure prediction tool AlphaFold2 to assess the variant pathogenicity. The p.Cys800Ser variant has an intermediate score that falls into the VUS category (Table 1) rather than a definitive benign or pathogenic classification. Therefore, cell-based studies were needed to gather more information and allow the variant reclassification.

### 3.2. Establishment of Stably Transduced NPC1^−/−^ ARPE-19 Cell Lines

**Figure 2:**
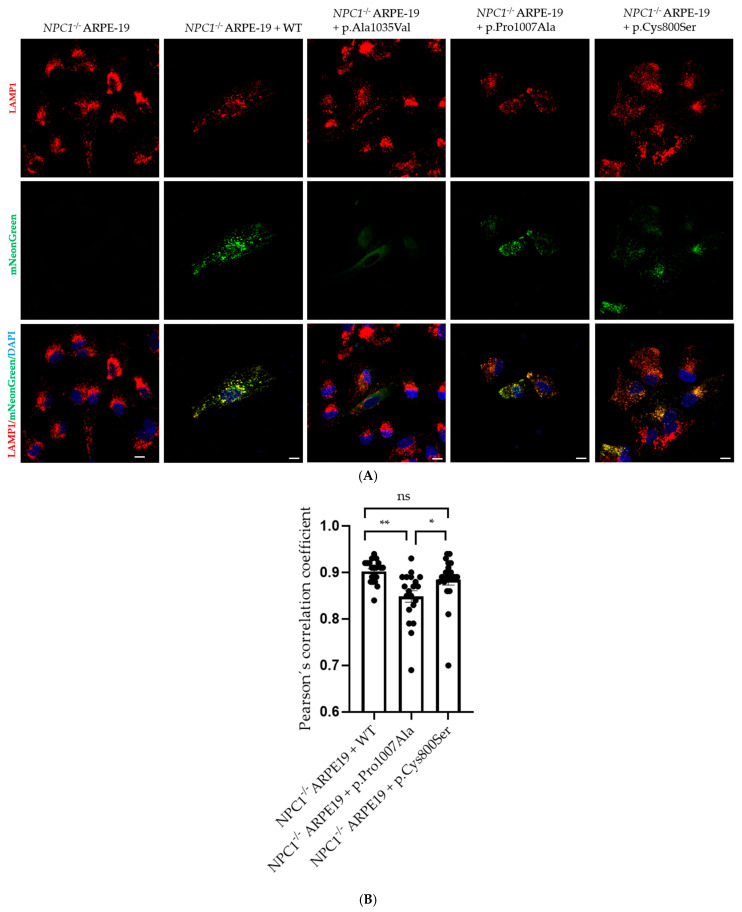
(**A**) Colocalization of LAMP1 and NPC1-mNeonGreen in NPC1^−^/^−^ ARPE-19 cells. From left to right, images show NPC1^−^/^−^ ARPE-19 cells that are either non-transduced (NT) or transduced with NPC1 wild-type (WT), a known pathogenic variant (p.Ala1035Val or p.Pro1007Ala), or a variant of uncertain significance (p.Cys800Ser). White scale bar: 25 µm. (**B**) Colocalization measurement of LAMP1 with mNeonGreen in transduced NPC1^−/−^ ARPE19 cells. Various regions of interest were selected for each cell line and 20 regions of interest were analyzed; the Pearson’s correlation coefficient was calculated for each region. Comparisons between groups were done using Tukey’s multiple-comparison test (** *p* < 0.01; * *p* < 0.05).

Genetically modified *NPC1*^−/−^ ARPE-19 cells have been shown to faithfully mimic the cellular characteristic of NPC pathology [8]. Being aware of their efficiency of transduction, the cells were infected with γ-retroviral particles produced in-house. These particles carried the different NPC1 variants of interest cloned into a pBABE-puro vector, in which the NPC1 cDNA sequence was fused to the coding region of mNeonGreen protein. Successful rescue of the NPC cellular phenotype was observed in transduced cells with the WT NPC1 construct, confirming the functionality of the exogenous protein. Additionally, mNeonGreen-tagged NPC1 expression was confirmed across all transduced cell lines via fluorescence microscopy. As expected, the NPC1-WT appears primarily in a punctate pattern, consistent with the late endosomal/lysosomal distribution of endogenous NPC1 (Figure 2, second panel). Instead, cells transduced with p.Ala1035Val exhibited a diffuse pattern resembling a misfolded protein, retained in the ER and not able to reach the late endosome/lysosome compartments (third panel). Concerning p.Pro1007Ala, the punctate pattern is also observed (last panel), which is consistent with a mild variant (associated with a late-onset clinical form).

### 3.3. NPC1 Variant p.Cys800Ser Is Transported to the Lysosome

Immunofluorescence assays on NPC1^−/−^ ARPE-19 cells transduced with the different mutants revealed that, like the WT protein and p.Pro1007Ala, the p.Cys800Ser variant reaches the lysosome since it colocalizes with the LAMP1 protein (red) (Figure 2, last panel). The representative images displayed in Figure 2 show the merging of the NPC1-mNeonGreen signal (green), the LAMP1 signal (red) and the DAPI signal in the transduced cells. Conversely, p.Ala1035Val does not reach the lysosome, as expected (third panel), matching with the severe phenotype reported in the homozygous patients for this variant.

### 3.4. NPC1 Variant of Unknown Significance p.Cys800Ser Presents Expression Levels Similar to p.Pro1007Ala

**Figure 3:**
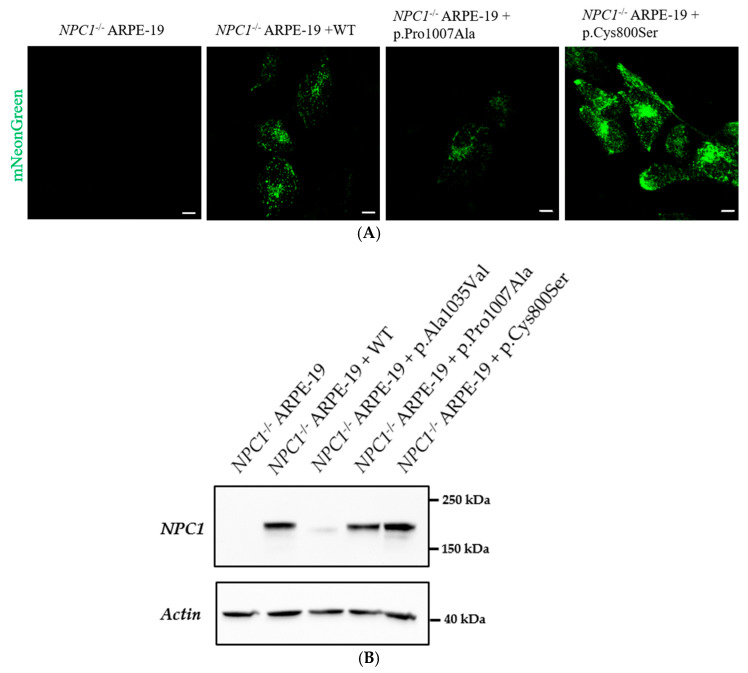
(**A**) NPC1-mNeonGreen expression in NPC1^−^/^−^ ARPE-19 cells and in transduced NPC1^−^/^−^ ARPE-19 cells. From left to right, images show NPC1^−^/^−^ ARPE-19 cells that are either non-transduced (NT) or transduced with NPC1 wild-type (WT), p.Pro1007Ala or p.Cys800Ser variants. Representative images for both are shown. White scale bar: 25 µm. (**B**) Representative Western blot showing NPC1 protein levels in NPC1^−/−^ ARPE-19 cells (no protein, as expected) and transduced cells with either the wild-type (WT), the p.Ala1035Val, the p.Pro1007Ala or the VUS p.Cys800Ser variants.

The created cell lines were screened for expression of mNeonGreen by fluorescence microscopy and the NPC1 protein levels in the transduced cell lines were assessed by Western blot. NPC1-mNeonGreen expression in NPC1^−^/^−^ ARPE-19 cells transduced with the WT and Pro1007Ala variants displayed the expected cellular phenotypes, and the ones transduced with the p.Cys800Ser-mNeonGreen construct also presented a punctate pattern similar to the wild-type and p.Pro1007Ala variants but with a slight, visually apparent difference in expression level in both immunofluorescence and WB experiments (Figure 3).

### 3.5. NPC1 Variant of Unknown Significance p.Cys800Ser Induces Changes in Lysosome Positioning When Compared to WT-NPC1

Proper lysosomal function relies heavily on accurate positioning and dynamic motility, both of which are regulated by intricate cellular mechanisms. A hallmark of many LSDs, including NPC, is the abnormal centripetal accumulation of lysosomes [16,17], tending to form an increased number of membrane contact sites with the ER, which significantly restricts their mobility and consequently impairs their functional capacity.

**Figure 4:**
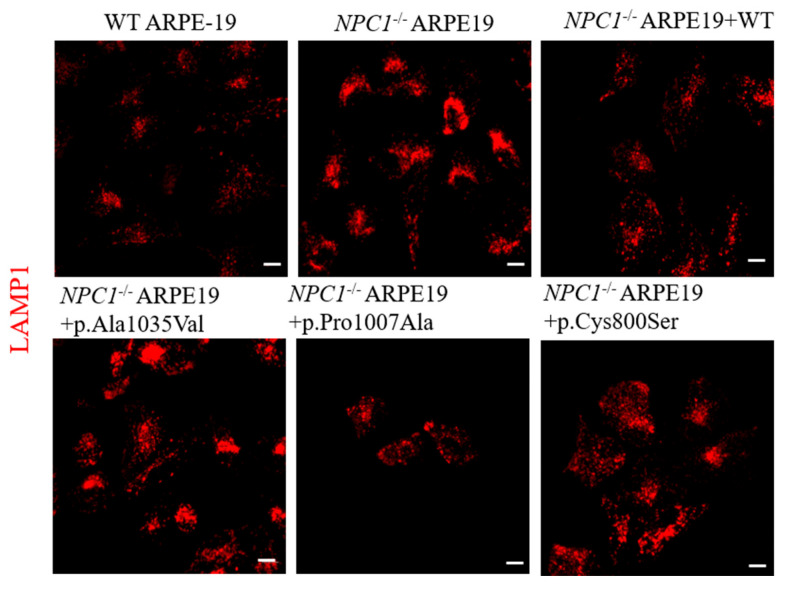
Lysosomal distribution in ARPE-19 and NPC1 ^−^/^−^ ARPE-19 cells. The first image (upper panel) shows the LAMP1 signal (red) in WT ARPE-19 cells; the other images show NPC1^−/−^ ARPE-19 cells under different transduction conditions: non-transduced (NPC1^−/−^ ARPE-19), transduced with NPC1 wild-type (NPC1^−/−^ ARPE-19 + WT), with a pathogenic variant (NPC1^−/−^ ARPE-19 + p.Ala1035Val or NPC1^−/−^ ARPE-19 + p.Pro1007Ala), or with a variant of uncertain significance (NPC1^−/−^ ARPE-19 + p.Cys800Ser). Representative images for both are shown. White scale bar: 25 µm.

LAMP1 staining in the analyzed cell lines showed altered lysosome positioning in the NPC1^−/−^ ARPE-19 cells when compared with the ARPE-19 WT cells, as expected (Figure 4, first and second panel). Upon transduction with the WT NPC1 construct or the p.Pro1007Ala mutant, the lysosomes acquired a normal distribution (Figure 4), but with the mutant p.Ala1035Val, clustering of the lysosomes in the perinuclear region was observed.

Concerning the analyzed VUS p.Cys800Ser variant, the lysosomes acquired an intermediate distribution between p.Pro1007Ala and the p.Ala1035Val (Figure 4, last panel).

## 4. Discussion

The *NPC1* gene is highly polymorphic, with over 810 variants associated with disease identified to date (assessed on HGMD database on 9 January 2026). A large number of NPC patients are compound heterozygotes, often carrying private mutations. Other variants are recurrent globally or among distinct populations. When a novel variant in the *NPC1* gene is identified upon clinical suspicion, many studies need to be carried out to disclose its pathogenicity. This is not always easy, especially in the case of missense variants.

This gap in knowledge negatively affects the clinical practice of genetic information, causing anxiety for VUS carriers or for individuals in compound heterozygosity with a pathogenic variant. Leveraging technology, some in silico models can be used to predict the pathogenicity likelihood of genetic variants. Also, many recommendations that provide a standardized framework for interpreting sequence variants in clinical genetic testing, known as American College of Medical Genetics and Genomics (ACMG) guidelines, are applied.

However, additional experimental evidence is imperative to determine if each VUS is benign or pathogenic. Thus, it is important to create new experimental systems to address the pathogenic potential of VUS in a personalized way. Of note, patient-derived skin fibroblasts are a well-established model for studying NPC pathology and have frequently been used to examine the intracellular trafficking and degradation pathways affected by various NPC1 missense mutations [18]. Nonetheless, these in vitro systems present certain limitations; notably, fibroblasts undergo senescence after a limited number of passages, compromising reproducibility and invoking ethical dilemmas. Secondly, this system does not allow the study of the effect of only one variant in the case of compound heterozygosity.

Furthermore, the limited availability of high-quality antibodies against endogenous NPC1 hampers their use in immunofluorescence-based assays. To overcome these challenges, we generated stable cell lines expressing wild-type (WT) NPC1, as well as the variants mentioned, each tagged with mNeonGreen. These engineered cell models were used to examine variant-induced alterations in NPC1 protein trafficking, as well as their effects on lysosome spatial distribution. Of note, this cell model is only suitable for missense variants.

In this context, we developed and applied a set of tools to investigate a VUS in the *NPC1* gene: p.Cys800Ser. Our goal was to determine whether this variant is pathogenic or benign. Although some cellular alterations were observed in the stable cell line expressing the p.Cys800Ser variant, the interplay between the two variants identified in the patient reported here (p.Ala521Ser and p.Cys800Ser) and the microdeletion (del15q11.2) contribute to a unique combination of clinical symptoms and increase the awareness for the complexity of NPC disease, specially the juvenile and adult forms. Noteworthy, the clinical symptoms that the patient presents can evolve over time, since the p.Cys800Ser variant does not impair the intracellular trafficking of the NPC1 protein to the lysosome, which is also occurs in other mild NPC1 variants that are associated with the adult clinical form of NPC disease when present in homozygosity. This is the case for patients homozygous for the p.Pro1007Ala variant, for instance [7,19].

## 5. Conclusions

Taking into account the importance of classifying novel genetic variants into pathogenic or benign, and considering that in some cases in silico tools are not predictive, it is imperative to create good cellular models to assess/evaluate their effect in vitro/experimentally. In this work, we leveraged NPC1^−/−^ ARPE-19 cells, which recapitulate cellular mechanisms of NPC disease, and created stable cell lines with γ-retroviral particles carrying the different NPC1 variants.

Confocal analysis showed significant differences between different pathogenic variants. Indeed, the well-known pathogenic variant p.Ala1035Val led to the retention of NPC1 protein in the endoplasmic reticulum, and it does not reach the lysosome, as expected [8]. Conversely, the NPC1 protein harboring the ‘milder’, yet still disease-causing, p.Pro1007Ala variant had a profile similar to WT, which is consistent with the adult form of the disease. For the VUS p.Cys800Ser, it elicits a WT-similar pattern concerning its transport to the lysosome, even though the lysosome distribution in cells transduced with this mutant was different from that seen in WT and p.Pro1007Ala models. Of note, lysosomal distribution/positioning correlates with NPC1 pathogenicity and consequently severity of the clinical phenotypes. Indeed, this was observed in our previous publication [8] in which lysosomal clustering was surveyed in the NPC1^−/−^ ARPE-19 cell lines transduced with different variants. We observed that the variant associated with a more severe phenotype (p.Ala1035Val) displayed higher lysosomal clustering than another disease-causing variant associated with a less severe phenotype.

This seems to indicate that the variant reported here, p.Cys800Ser, might be related to lysosomal dysfunction, but further studies are needed to confirm its pathogenicity. Additionally, the stable lines created are suitable for future transcriptomic, proteomic, and lipidomic studies, as well as for testing new drugs or repurposing existing ones. In summary, while not fully disclosing our target variant’s pathogenicity, this work has enabled the development and characterization of novel, biologically relevant cellular models and contributed to advancing the functional characterization of *NPC1* gene variants, with possible impacts on improving the molecular diagnosis of NPC patients and reducing the diagnostic odyssey that they often face.

## Figures and Tables

**Table 1:** AlphaMissense prediction scores (https://alphamissense.hegelab.org/search, accessed on 15 January, 2026) for the VUS p.Cys800Ser and, as comparison, for the already described pathogenic variants p.Pro1007Ala and p.Ala1035Val. REVEL Pathogenicity scores for the variants p.Pro1007Ala and p.Ala1035Val were not available.

AlphaMissense			
Variant	Classification	AlphaMissense Score	REVEL Pathogenicity Score
p.Cys800Ser	VUS	0.491 Ambiguous	0.91 Likely pathogenic
p.Pro1007Ala	Pathogenic	0.5984 Likely pathogenic	--
p.Ala1035Val	Pathogenic	0.6819 Likely pathogenic	--

## Data Availability

Data from this study are available upon request to the corresponding author.

## Abbreviations

The following abbreviations are used in this manuscript:

NPC	Niemann–Pick type C
VUS	Variant of unknown significance
ARPE-19	Arising retinal pigment epithelia 19 cell line
ACMG	American College of Medical Genetics and Genomics
WT	Wild-type
LAMP1	Lysosome-associated membrane protein 1
DAPI	4′,6-diamidino-2-phenylindole
CGH	Comparative Genomic Hybridization
